# Developing a Dynamic Model for Assessing Green Infrastructure Investments in Urban Areas

**DOI:** 10.3390/ijerph182010994

**Published:** 2021-10-19

**Authors:** Esther Barrios-Crespo, Saúl Torres-Ortega, Pedro Díaz-Simal

**Affiliations:** 1 Civil Engineering School, Universidad de Cantabria, 39005 Santander, Spain; esther.barrios@alumnos.unican.es; 2 IHCantabria—Instituto de Hidráulica Ambiental de la Universidad de Cantabria, 39011 Santander, Spain; pedro.diaz@unican.es

**Keywords:** system dynamics, green and blue infrastructure, ecological restoration, profitability analysis, urban modelling

## Abstract

In recent decades, cities have been experiencing accelerated population growth, associated with an increase in the scales of production and consumption. This fact, combined with deficient management of resources and waste, has led to the loss of biological diversity, compromising the generation of ecosystem services, with disastrous consequences for human health and well-being, but also for the economic system. In the field of civil engineering, the predominance of artificialisation and impermeabilization of cities (called “grey engineering”) is being questioned to be replaced or complemented with new types of infrastructures that represent a transformative change to achieving more sustainable cities. Through system dynamics applied to the economic modelling of the city of Santander (Spain), the aim of this study is to analyse the profitability of investment in ecosystem restoration and in both green and blue infrastructure, and of the implementation of environmental policies based on the relationships of affection established in the model, which represent the interactions between the main actors in urban dynamics. As a main conclusion, it is found that investing in green infrastructures and ecosystem restoration, and environmental policies is highly profitable: EUR 1 spent can produce up to EUR 100 as a benefit.

## 1. Introduction

Currently, more than half of the world’s population lives in cities, and by 2030, this figure is expected to reach 60% [1,2]. Cities are complex ecosystems governed by socio-economic activities and natural processes simultaneously, and therefore urban ecosystems need integrated, effective, comprehensive, and multifunctional ecological infrastructures [3,4]. The vertiginous process of urbanisation in recent decades has meant that the decisions of the human population living in cities affect the resilience of the entire planet [5]. The urbanisation of cities faces fundamental challenges, but also an unprecedented opportunity to achieve resilience and ecological functioning of urban systems [6].

As a consequence of current population growth, cities have experienced, over the last 50 years, an increasing demand for services such as energy, food, water, materials and land use, which has placed a significant cost on planetary systems [7].

The increasing production from non-renewable resources generates new global challenges such as climate change and biodiversity loss [8]. According to the World Economic Forum’s Global Risks Perception Survey [9], five of the top ten most likely major risks facing society today belong to the environmental category: extreme weather, failure of climate action, natural disasters, loss of biodiversity and anthropogenic environmental disasters.

Current trends of excessive global population growth suggest that by 2050, the Earth’s land area free from the impact of anthropogenic activities will have shrunk to one-tenth of the planet [10]. Climate change, land-use change, ecosystem fragmentation and habitat loss, overexploitation of natural resources, high levels of pollution and the presence of invasive species are the main threats to biodiversity in today’s cities [11].

According to the Millennium Ecosystem Assessment, most of the actions developed in the past decades to attenuate or reverse the degradation of ecosystems have yielded significant benefits; however, these improvements have generally not kept pace with growing pressures and demands [12].

Both problems, the growth of urban population and the need to preserve ecosystems, show the relevance of cities as emerging areas where further study on the correct management of nature areas is clearly needed.

The relationship between ecosystem services and human quality of life is revealed through the influence that ecosystem disturbances have on the various components of human well-being, such as the basic material needs for a good life, health, good social relations, security, and freedom of choice and action, in addition to their significant contribution to global employment and the economic activity [12].

Nature-based solutions, which imply bringing nature back into cities, can include retrofitting green and blue infrastructure, creating and conserving green spaces and water bodies, promoting urban agriculture, implementing sustainable urban drainage systems, and providing ample and accessible vegetation cover in urban and peri-urban areas. According to [13], increased use of green infrastructure and other ecosystem-based approaches advances the development of urban sustainability, while also serving to strengthen climate mitigation and adaptation. Furthermore, as defined by the European Union, nature-based solutions should be “cost-effective, simultaneously provide environmental, social and economic benefits and help build resilience” [14].

The design and management of this green infrastructure aim to harness nature’s self-capacity to provide the full range of ecosystem services and the protection of urban and rural biodiversity [15]. Several studies such as the one developed by Elmqvist et al. [6] show that increased investment in green infrastructure in the urban landscape can be a potential source of both monetary and non-monetary benefits for society and the common good, contributing to the conservation of biodiversity and the development of more resilient urban areas. The presence of trees, parks and gardens, wetlands, green roofs, forests and other natural spaces increases the generation of ecosystem services, improving the urban environment, contributing to the mitigation of the effects of climate change and maintaining ecological balance [16]. Some research has found that the cost of investing in green infrastructure can be considerably lower than traditional grey infrastructure, whilst highlighting its multi-functional nature and lower maintenance requirements [3]. 

Ecological restoration, considered as the process by which the re-establishment of an ecosystem that has been degraded, damaged or destroyed is promoted [17] is aimed at optimising biodiversity and ecological processes, and the generation of ecosystem services, taking into account the ecological, socio-economic and cultural framework. According to [18], the multiple benefits of ecosystem restoration include protection against extreme meteorological events, prevention of erosion, carbon sequestration, habitat restoration, etc. Moreover, according to this report, the benefits of ecosystem restoration are likely to outweigh the costs of implementation, particularly in the case of wetland, grassland, inland and coastal forest restoration [18,19]. Other recent studies estimate the cost of environmental impacts of human activities in trillions of US dollars annually in loss of goods and services [20]. Although proper maintenance, conservation and the sustainable use of biodiversity and ecosystems have been stated to be cheaper, given the present state of ecosystem degradation, restoration is currently considered an imperative [21].

While multiple studies identify and highlight the importance of urban ecosystem services and natural capital and the environmental threats they face [22,23], fewer studies address the understanding of the complex dynamics of ecosystems in urban environments. In this group, the most relevant examples apply a system dynamics approach [24,25,26], and particularly, in terms of the economic impact of restoring these services through investment in green infrastructure and ecosystem restoration [6,19]. It is crucial to emphasise the importance in this study of economic quantification and valuation of ecosystem services and natural capital, beyond the mere ecological criteria [22,27,28,29] introducing the concept of ecological economics [30,31].

The main objective of the work presented in this article is to test the favourable influence of increased investment in nature-based solutions on the economy and the quality of life of the inhabitants of the city of Santander (Spain), by means of a system dynamic model.

Although the development of a dynamic model of a city is not an entirely novel idea [25,32,33,34,35], the idea of using it to study the effects of investment on green infrastructure, as a solution to the environmental threats that cities face today, is a more recent approach, which is not sufficiently settled in the literature [24,36,37,38]. For this purpose, in this study, a cost-benefit analysis of investment nature-based solutions has been carried out, as well as the implementation of new environmental policies (i.e., reduction of pollutant emissions, waste management, etc.).

## 2. Materials and Methods

The following sections aim to present the data used in the elaboration of the economic system dynamics modelling of Santander, as well as to describe the methodological process that has been carried out for its elaboration. First, a short description of the software used is presented. Then, the study area to be modelled using VENSIM and whose urban dynamics are to be analysed is described. Lastly, the theory of system dynamics (SD) is introduced, with a detail description of the economic SD model of Santander, the development of which is presented later.

### 2.1. VENSIM Software

The VENSIM PLE (Ventana Systems Inc., Harvard, MA. USA) software by Ventana Systems is a simulation software that allows the set of equations that make up a complex dynamic system to be easily solved. VENSIM is a widely used system dynamics modelling system for simulation applications due to its intuitive user interface. In addition, VENSIM offers a flexible way to dynamically visualise and communicate the complexity of how systems and ideas work by constructing a wide variety of simulation models from causal loops or flow and balance diagrams [39].

System dynamics software have broadly expanded in the last 20 years. The selection of VENSIM as analytical framework is based on four reasons: first, its computational efficiency is out of discussion; second, the accessible PLE version allows easily replicating the model for further research; third, the simplicity of the interface invites to easily adopt it for newcomers; and finally, the existence of a dynamic community that supports open training resources invites to use it as a reference. These circumstances are not unique for VENSIM but globally observed VENSIM is well ranked in all of them.

### 2.2. Study Area

The city of Santander is the capital and the main population centre of the province of Cantabria (Spain). This study area has been selected as case study for several reasons. First, coastal cities are settled in direct connection with coastal environment. Therefore, the urban density—environmental quality—urban conditions feedback loops are visible and accessible for the society subject to it. The decision-making process will be sensible to the situation when, for example, beaches are affected by pollution and individuals cannot enjoy from them. 

Second, even when climate change is involved (and the impact is not direct), coastal cities are good examples of exposed areas to environmental impacts. In this sense, the situation of Santander, located in a coastal area, near a cape and creating a bay, creates space scarcity that generates social debates on how to handle the emerging issues as the “send it further” option does not exist in this case.

Lastly, the reasonable size of the municipality and the availability of data [40,41] for the analysis support the decision to focus the analysis on Santander.

**Figure 1:**
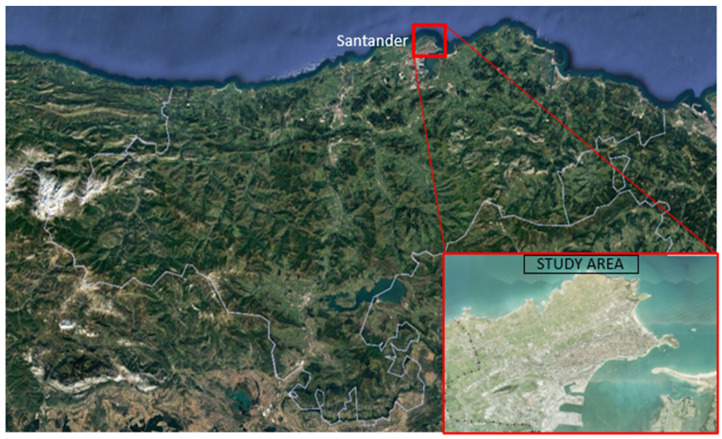
Location of the study area in the region of Cantabria (Spain) and delimitation of its borders (own elaboration).

Based on these, the study area was considered to be the municipality of Santander itself and, and the surroundings of the Bay, partially made up of the localities of Somo and Pedreña (Figure 1). This area represents up to 35% of the inhabitants of Cantabria [42].

The economic structure of the study area is closely linked to its geographical location, between the bay and the Cantabrian Sea. It is for this reason that the economic development of the city of Santander is parallel to the development of its port and the urban area of the city [40]. Around its port, the growth of maritime transport of goods has taken place with an upward trend from 1962 to the present day [43] and passengers, highlighting its regular shipping lines with connections to the United Kingdom and Ireland, and the local shipping lines between the shores of the bay.

According to data from the CORINE Land Cover [44], 85.65% of the study area is made up of water bodies; 10.8% is agricultural land; artificialized land comprises 3.05%, while the urban fabric covers 1.95% of the studied area; and the land area occupied by forests and semi-natural areas, and urban green spaces accounts for only 0.5% of the total area analysed. The percentage of natural and semi-natural areas is a minority (0.41% of the study area), as are urban green areas (0.09%). Concerning the total urban area of the city of Santander, the percentage of urban green areas is only 4.7%, being the remaining 95.30% built-up and impervious areas, while regarding the total artificialized area, the percentage of green spaces is only 2.95%.

Although the bay area includes a site of community importance (SCI) located on the eastern shore, called ES 1300005 Dunas del Puntal and Estuario del Miera [45], and a special protection area for birds (Isla de Mouro) [46], both included in the Natura 2000 Network, the bay is surrounded by a wide range of anthropogenic activities, with consequent effects on the bay’s ecosystem.

The study area must cope with several risks affecting both the physical, natural and socio-economic environment. The Bay of Santander has suffered for decades from the high levels of pollution generated in the inner part of the bay from the predominant industrial activities. At present, despite a decrease in pollution levels as a result of the ceasing of some of these activities, there are still many problems that require management. The most relevant issues are the control of ballast water from ships that frequent the bay, which is one of the main sources of invasive species, and pollution derived from the use of heavy hydrocarbons in navigation [43].

For the identification of the environmental units with an influence on the study area, the area of the Bay of Santander has been considered. This area includes environmental units identified as ecosystem services’ providing elements capable of providing the population with resources are broadleaved forests, heathlands, and areas of sparse vegetation; crop fields and areas used for agriculture; coastal ecosystems: beaches, dunes and cliffs; wetlands and estuaries; meadows and pastures.

From an environmental perspective, the bay and its surroundings are an important source of resources and ecosystem services, both for supporting, provisioning, regulating and cultural services (Table 1 and Table 2). Among the provisioning services, the bay stands out as a habitat for a multitude of edible species: fish, shellfish, crustaceans that not only serve as food for humans but also other fish and poultry species. Conversely, grasslands and pastures are the main sources of food for livestock species, from which food for human consumption such as dairy or meat products are obtained. In addition, animal materials such as leather and wool are obtained from livestock farming. Meanwhile, the main purpose of croplands (predominantly small agricultural areas scattered throughout the territory, such as orchards, nurseries, and intensive crops [47] is to supply food through the priority cultivation of vegetables and fruit. In terms of regulating services, the environment of the Bay of Santander has the potential to contribute to the city’s climate regulation, as large bodies of water such as wetlands and estuaries are important carbon sinks, as well as the surrounding vegetation that contributes to carbon sequestration and storage. Consequently, these ecosystems offer the capacity to absorb atmospheric pollutants, leading to an improvement in air quality. Forests, heathland, and grasslands present in the area allow the regulation of the hydrological cycle, favouring water infiltration and the recharge and maintenance of aquifers, while favouring evapotranspiration, preventing soil erosion, and playing a fundamental role in the prevention of flooding caused by extreme weather events. Forests, grasslands, and riparian vegetation, as well as the crops present in the study area, play an essential role as food for pollinating species, providing an optimal environment for the promotion of pollination. It is also worth highlighting the function of the ecosystems around the bay as a habitat for a wide variety of animal and plant species. The ecosystems present also have a high value in terms of the cultural services they offer, not only due to the high landscape value of places such as the Bay of Santander itself, the beaches of El Sardinero, the Menor and Mayor capes, or the peninsula of La Magdalena, among others, but also for the potential recreational use of these natural areas.

### 2.3. Dynamic Modelling

System dynamics (SD) is a well-established system simulation methodology introduced by Jay Forrester in the 1960s, aimed at understanding, visualising, and analysing complex dynamic feedback systems [48]. Through SD, it is possible to analyse a set of cause-effect relationships between the different factors involved in the system and, through computer simulation, to perform a quantitative analysis of the structure of the information feedback system and the dynamic relationship between the variables and the behaviour of the system [25].

In a system dynamics analysis, it is essential to understand the interactions among many related social, economic, environmental, managerial, regulatory and lifestyle factors [24]. The difficulty of these interactions lies both in their simultaneous effect on various components of the system, and in their temporal variation. System dynamics is considered a useful tool for predicting and analysing the outcomes that result from the interactions between system components, and for analysing the implications of policy implementation.

#### 2.3.1. System Dynamics Modelling of Santander

**Figure 2:**
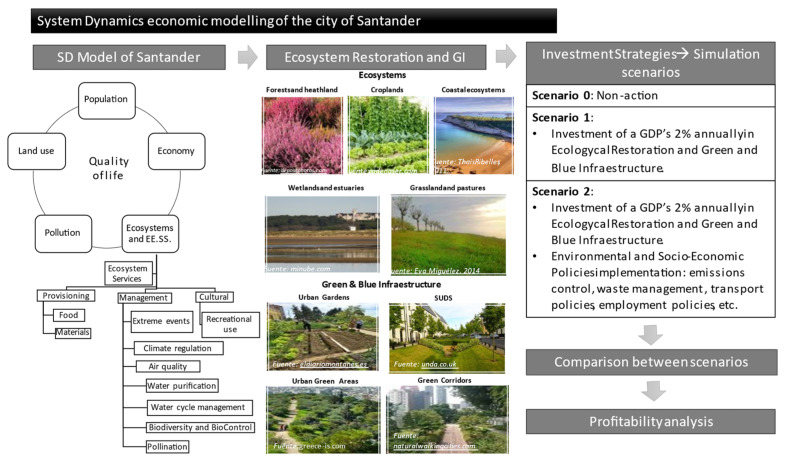
Schematic diagram of the steps in the methodology followed in this study. It illustrates the main components of the SD model of the city of Santander and emphasizes the principal ecosystems and GI elements present in the study area to consider in the modelling process. It also states the different scenarios simulated representative of the investment strategies considered, and the following comparison and analysis of the profitability of the investment and policies proposed (own elaboration).

The SD model of Santander and the Bay Area comprises five main systems that constitute the principal dimensions involved in urban dynamics (Figure 2). Each of these systems and the set of variables and relationships that constitute them are shown individually below.

**Figure 3:**
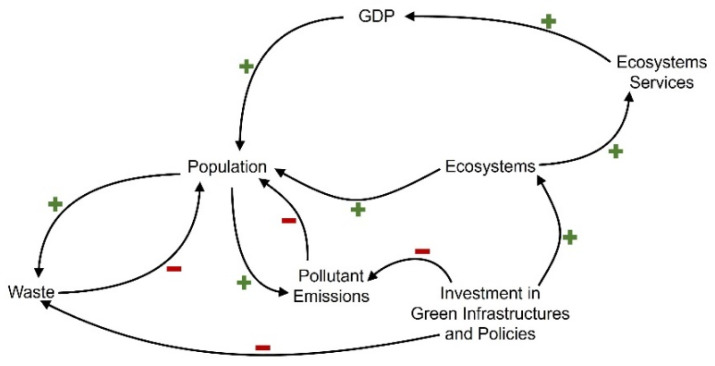
Casual loop diagram of the main model systems (own elaboration).

The main logic of the model is represented in Figure 3. This figure represents the casual loop for the main systems of the model. An increase on population translates into an increase on emissions and waste production, which implies a reduction of population by the increase of diseases. On the contrary, better and more ecosystems implies a better environment for the population. Investment on green infrastructure and policies translate into a reduction of pollutants and waste, and an increase of ecosystems, which derives into an increase of GDP (via more ecosystem services), which can be assumed to be a good indicator for population quality of life.

##### Population System

The population of the study area is modelled through a level variable that represents the balance between the flows of births and deaths in the city, dependent on the respective birth and death rates. Moreover, since the population dynamics of a city are dependent on multiple variables which, in turn, depend on other factors, a wide number of auxiliary variables with an effect on the temporal behaviour of the population in the study area and vice versa has been included.

**Figure 4:**
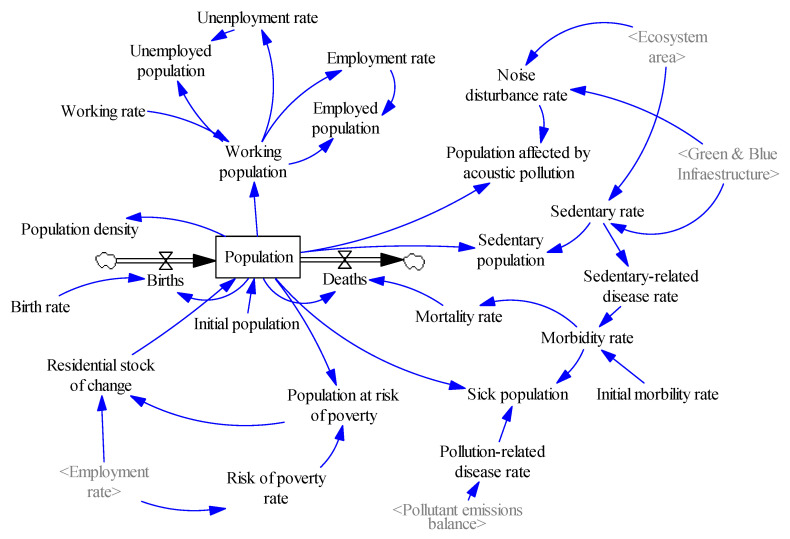
Population system (own elaboration).

As shown in Figure 4, the mortality rate is related to the morbidity rate, and this depends on the sedentary-related disease rate (linked with the disposal of natural spaces) and on the pollution-related disease rate (linked with the pollutant emissions balance). Moreover, the population balance is also affected by the residential stock of change, dependent on the risk of poverty rate, being this variable inversely proportional to the employment rate of the city.

##### Pollution System

**Figure 5:**
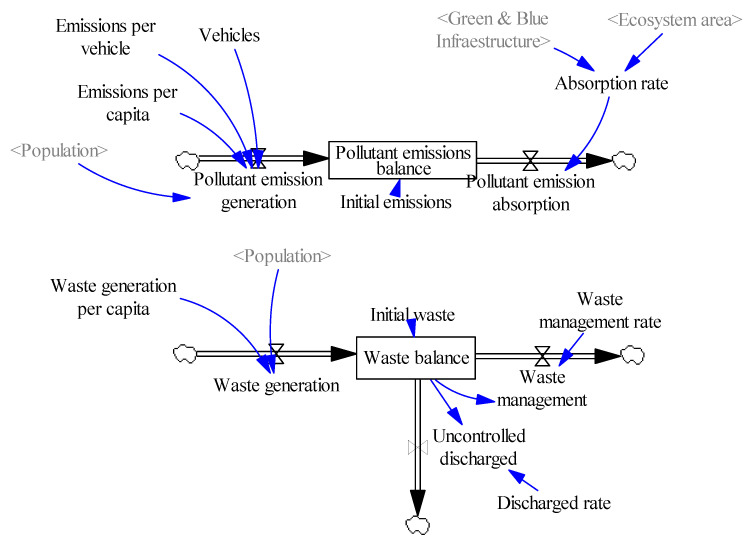
Pollution system (own elaboration).

As a fundamental part of the model, it was considered essential to analyse the effects of pollution on the development of the city’s ecosystems, population, and economy. The main sources of pollution in cities have been considered to be the balance of pollutant emissions and, conversely, uncontrolled waste dumping. Each of these sources of pollution has been represented as an individual system by Forrester diagrams (Figure 5).

Pollution generation depends on emissions per capita and emissions per vehicle. This distinction has been made according to the source of emissions to be able to analyse afterwards the effects of, for example, the reduction of transport by private cars. Conversely, the absorption of pollution depends on the absorption rate of the system, depending, in turn, on the absorption capacity of ecosystems present in the area.

The balance of greenhouse gas pollutant emissions is represented as tonnes of CO_2_ equivalent, based on data from the Air Emissions Accounts [49]. Nevertheless, the total emissions accounted for are made up of different types of pollutants such as nitrogen oxides (NO_X_), sulphur oxides (SO_X_), methane (CH_4_), PM2.5 and PM10 particles, volatile organic compounds (VOC), carbon dioxide (CO_2_), etc.

The pollution resulting from the production and dumping of waste has been modelled based on a level variable, waste pollution, which receives the flow coming from the generation of waste, calculated as the waste per capita per population of the study area, of which a part is destined to waste management, determined by the waste treatment rate, and another part is made up of uncontrolled dumping into the environment, which depends on the dumping rate.

##### Area Distribution System

**Figure 6:**
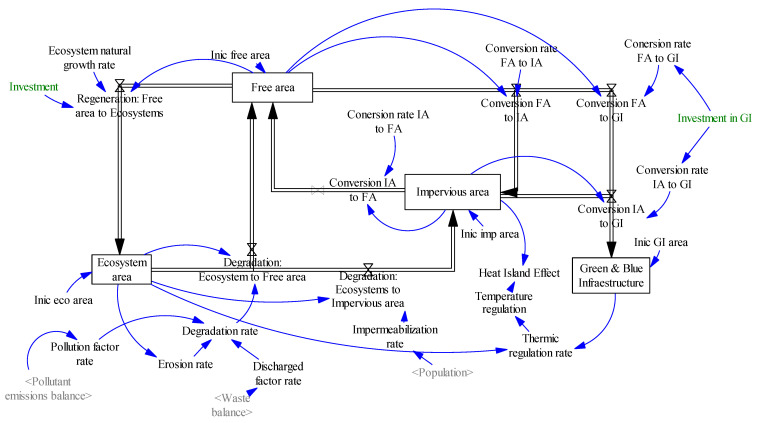
Areas distribution system (own elaboration).

A system has been developed that establishes the relationships between the areas occupied by ecosystems, green and blue infrastructure, impervious surface, and free surface (Figure 6). Each of these surface typologies is modelled as level variables, which receive inflows or outflows that imply a growth in the surface area occupied by each one, or its degradation or transformation into another typology. These transformations are governed by the respective rates of degradation, impermeabilization, etc., which in turn, depend on other systems participating in the model, such as pollution levels, population, etc.

##### Ecosystems and Ecosystem Services System

**Figure 7:**
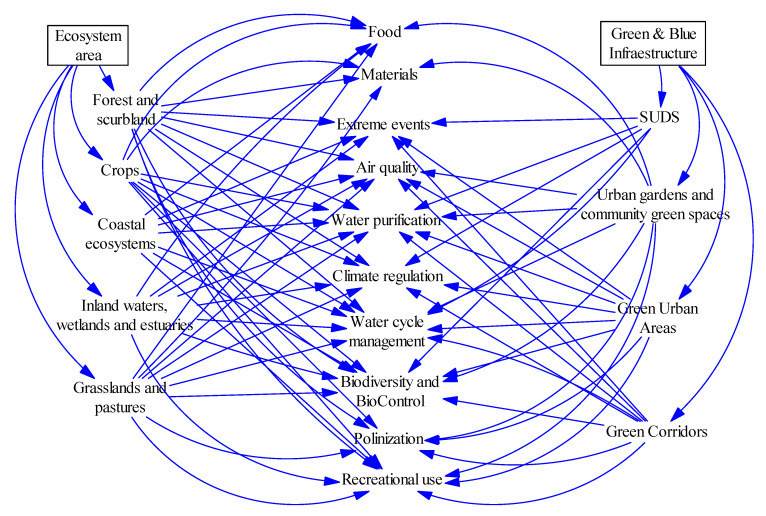
Ecosystems and ecosystem services system (own elaboration).

The area of ecosystems shown in the Forrester diagram of area distribution can be subdivided according to the type of ecosystem it occupies in forests and shrublands, crops, coastal ecosystems, inland waters, wetlands and estuaries, and grasslands and pastures (Figure 7). The area occupied by green and blue infrastructure is also classified into the following categories: sustainable urban drainage systems; urban gardens and community green spaces; urban green areas; and green corridors. Having defined the types of ecosystems and green infrastructure present in the study area and identified the ecosystem services they provide, they have been included in the model using auxiliary variables and parameters that represent the estimated economic value of the ecosystem service per surface area per month, for each of them, according to the database “The TEEB Valuation Database” drawn up by Van del Ploeg and de Goot [50].

##### Economy System

**Figure 8:**
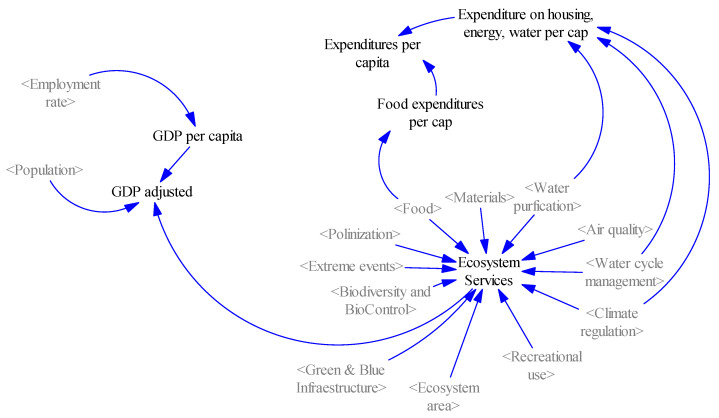
Economic system (own elaboration).

Finally, the economic dimension, represented by the gross domestic product variable, is introduced into the model (Figure 8). To model the influence of nature-based solutions on the city’s economy, a set of relationships have been established that simulate the effect of the variation of the surface area occupied by ecosystems in the city and its effect on the generation of ecosystem services and, finally, quantify the contribution of these services to the city’s economy, that is, to its GDP. Thus, we would be considering a GDP understood as a welfare function, in other words, a GDP adjusted by the economic contribution of ecosystem services.

The economic welfare indicator, as shown in the following equation:

GDP adjusted = GDP per capita × population + ecosystem services,

is calculated from the computation of direct GDP, and the economic benefits and savings derived from ecosystem services for families, quantified monetarily based on the values estimated by Van del Ploeg and de Groot [50].

Additionally, a direct relationship between GDP per capita and the employment rate has been considered, such that an interdependence is established between the variation in employability as a consequence of investments in nature-based solutions and the implementation of employment policies, and their repercussion on the per capita income of citizens.

Furthermore, the monetary quantification of the savings for families in the production of certain ecosystem services has been reflected in the variable “expenditures per capita”, which simulates, for example, the reduction in household food expenditure because of an increase in local food production, which would reduce the costs of acquiring the product, or the own cultivation in urban gardens, which would reduce this cost completely for certain products. Other considerations include energy savings in heating and air-conditioning due to the increased climate regulation capacity provided by ecosystems and green infrastructures.

#### 2.3.2. Investment and Policy Implementation Proposals

The investment and policy implementation proposals to be simulated in the alternative scenarios to the base case, and whose feasibility and cost-effectiveness—economically and in terms of quality of life—are to be analysed, as follows. First, according to the United Nations Environment Programme [51], “a green investment of just 2% of global GDP would be able to generate as much long-term growth in the period 2011–2050 as would occur under an optimistic business-as-usual scenario while reducing the negative impacts of climate change, water scarcity and the loss of ecosystems and their services”. Therefore, it is considered to invest 1% of Santander’s GDP per year in the regeneration of the city’s ecosystems and 1% in the implementation of green and blue infrastructure. 

In terms of environmental policies, a 55% reduction of pollutant emissions into the atmosphere has been established considering the objective of reducing CO_2_ emissions by 40–70% by 2050 set by the United Nations Environment Programme [51]. Conversely, to reduce pollution and the degradation of existing ecosystems due to uncontrolled waste dumping, it is established that 100% of the waste generated is treated.

Concerning policies related to transport and mobility, EU common policy principles based on the pursuit of the “sustainable mobility” model have been considered, particularly in the context of the sector’s growing green house gas (GHG) emissions, which constitute a threat to climate objectives. A 36% reduction in CO_2_ emissions from cars is therefore established. 

Finally, employment promotion policies have been established, increasing the employment rate by 10%, as an additional measure to the previous ones in the framework of goal number 8 of the 2030 Agenda for Sustainable Development “Decent work and economic growth”.

#### 2.3.3. Scenario Definition

To analyse different strategies and policies, three scenarios have been defined to be simulated within the model.

Scenario 0: The main assumption is zero investment in the regeneration and conservation of ecosystems and in the development and implementation of green infrastructure. This scenario represents the most unfavourable situation, and the consequences are likely to be negative for the system. 

Scenario 1: This second scenario assumes an investment in the restoration and conservation of ecosystems and the promotion of green and blue infrastructure in the study area. An investment of 2% of Santander’s GDP has been considered (following [51]), both in the regeneration of ecosystems and in the design and implementation of green infrastructure to create a resilient city capable of coping with the effects of climate change. For this purpose, the variables “investment in GI” and “investment in ecosystem restoration” have been introduced in the model.

Scenario 2: In the third simulation scenario, the effects of the application of a set of policies or measures affecting some of the fundamental parameters in the sustainable development of a city have been implemented into the system, together with the investments established in the previous scenario. The policies implemented are:

- Emission reduction policies: a constant reduction of 55% during the period of study has been considered, based on the UN’s [51], proposed target for the reduction of CO2 emissions by 40–70% between 2010 and 2050, aiming to be zero by 2070.
- Waste management policies: uncontrolled landfills: in order to reduce pollution from uncontrolled dumping of waste that is highly harmful to ecosystems, especially to bodies of water, the modifications to the simulation will consider a scenario in which 100% of the waste generated is treated, eliminating uncontrolled dumping into the environment.
- Employment policies: an increase of 10% of the employment rate is simulated, according to the eighth goal of the 2030 Agenda for Sustainable Development is “Decent work and economic growth”.
- Transport management policies: The number of vehicles has been reduced in a 25%, simulating the effects of pedestrianisation and other policies of this kind. Conversely, emissions derived from traffic have also been reduced in a 36%, according to the European Union’s common transport policy [52].

#### 2.3.4. Parameter Calibration and Validation

The main purpose of model calibration is to adapt the economic model of the city of Santander to the reality to be simulated, by adjusting the values of the different parameters, either by using real data or by using optimisation techniques (commonly used in empirical models). The values taken by the parameters and variables of the model after the calibration process must be consistent such that the model not only has a predictive value of reality for the set of data used in the calibration but also has an explicative capacity and the application of the model can be extrapolated to other scenarios. In addition, a validation process must be also carried out to verify that the model can be used to predict the future values of the parameters. Complementary, carrying out a sensibility analysis of the model parameters it is possible to assess the influence of the variation of certain parameters on the fundamental variables (i.e., the influence of a ±1% in the pollutant emissions in the GDP) and, therefore, to verify that the calibration of the model is satisfactory.

For the calibration of the population system, real data provided by the ICANE [53] for the 2000–2020 period has been used. The variables used includes total population, birth rates, mortality rate, population at risk of poverty rate and employment rate.

The active, unemployed, and employed population average values were calculated from the data provided by the INE [54]. It has been considered that sedentary lifestyles influence the evolution of labour activity, such that, with the increase in the sedentary population, the employment rate decreases, and the unemployment rate increases proportionally. 

For the sick population, the values used to calibrate ratios are based on data provided in the 2017 INE Hospital Morbidity Survey [55], considering that 20% of the main diagnoses are related to a sedentary lifestyle.

According to the Spanish National Health Survey [56] the sedentary population as a percentage of the total population is 44%. It has been considered that a greater availability of surface area occupied by ecosystems, natural areas and green and blue infrastructure can reduce this figure by up to half.

The representative pollution system has been calibrated based on emissions and waste generation data provided by the Air Emissions Account of the INE [57]. Emissions generated per capita and per vehicle have been distinguished for modelling separately, such that the influence of reduced vehicle use as a consequence of the implementation of transport policies, or the creation of green corridors can be taken into account.

For the modelling of waste pollution, waste per capita has been obtained from statistical data on waste collection and treatment provided by INE [58], with a value of 535.1 kg/person/year. It has been considered that 80% of the waste is destined for treatment, while the remaining percentage is uncontrolled dumping of this waste, which has been related to the implemented waste management policies.

The system modelling the different surface categories of the study area has been calibrated according to the initial area occupied by each category, which was defined based on data provided by CORINE Land Cover 2018 [44].

The erosion rate has been established considering that, according to the National Inventory of soil erosion [59], the erodible surface constitutes 96.16% of the surface area of Cantabria. If we disregard the percentage of area susceptible to zero erosion, and extrapolating this calculation to the study area, we could consider that the area of ecosystems susceptible to erosion in Santander is 91.91%, this being reduced by the erosion prevention effect exerted by the restoration and conservation of certain ecosystems.

The average GDP per capita in the municipality of Santander, calculated from data provided by the ICANE [60], is EUR 15,717.44 per person. A direct dependence between GDP per capita and the employment rate has been considered. The values for annual per capita expenditure have been obtained from the Household Budget Survey (HBS) data [61]. These data are EUR 3829.54/person for expenditure on housing, energy, and water and EUR 1,785.44/person for food, considering the average of the values for the time period 2006–2019. The 10% of expenditure on housing, energy and water has been found to vary inversely with the ecosystem services “climate regulation”, “water purification” and “management of the hydrological cycle”. 

##### Validation Results

**Figure 9:**
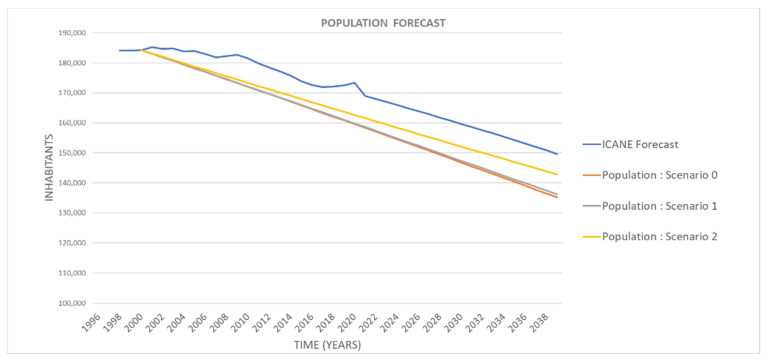
Comparison of the results of the model calibration and the population forecast offered by ICANE [62] (own elaboration).

Once the model has been calibrated, the reliability of the results obtained from the comparison of the demographic evolution in the modelled study area with the future projections (up to the year 2039) for the population of the municipality of Santander has been checked [62]. Figure 9 shows the results obtained in the model for the evolution of the population, influenced by the rest of the parameters involved in the simulation. It shows that population is satisfactorily adjusted to these predictions, with both showing a decreasing trend, and errors being less than 10%.

## 3. Results and Discussion

This section presents the results derived from the developed model. First, the results are presented and discussed, and second, an economic assessment (based on GDP estimates) to assess the profitability of implementing nature-based solutions is provided.

### 3.1. Comparison between Scenarios

The results obtained from the model are presented for the three simulation scenarios previously described representative of the different actions in terms of investment and implementation of economic, environmental, and social policies. The results obtained are represented in graphs showing the evolution of the main variables of the model, within the established time frame.

#### 3.1.1. Distribution of Surface Areas

First, representative graphs of the distribution of the surfaces of the study area are shown, in which the progression of the different surfaces can be seen. 

**Figure 10:**
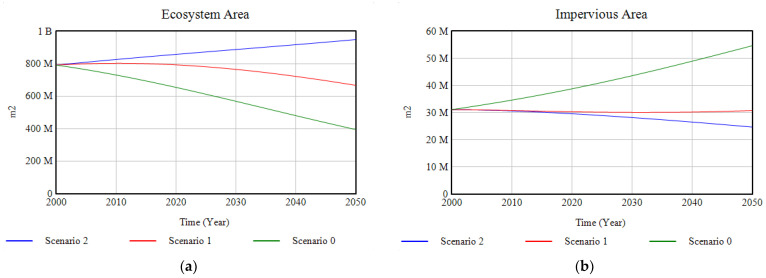
(**a**) Results of the modelling of the ecosystem area for the three scenarios; (**b**) results of the modelling of the imperious area for the three scenarios, in the period 2000–2050.

**Figure 11:**
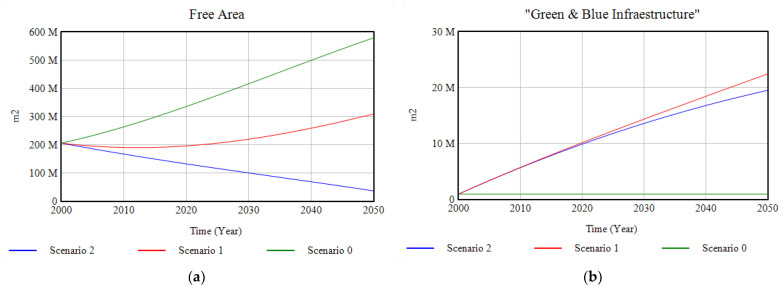
(**a**) Results of the modelling of the free area for the three scenarios; (**b**) results of the modelling of the green and blue infrastructure for the three scenarios, in the period 2000–2050.

As shown in Figure 10a, ecosystems, which presented a strong degradation in Scenario 0, soften their tendency to disappear through the annual investment of 2% of GDP in their restoration, and it is for Scenario 2, with the addition of a set of environmental policies, when their tendency becomes positive, experiencing a slight growth towards the year 2050. The evolution of the free surface area is complementary to that of the ecosystems, which means that part of this free surface area becomes part of the ecosystems through the proposed interventions. Conversely, the impervious surface (Figure 10.b), which undergoes a significant expansion in the base case, becomes relatively constant through investment in green and blue infrastructure, and for Scenario 2, it decreases, being replaced by urban green infrastructure (Figure 11).

#### 3.1.2. Population

**Figure 12:**
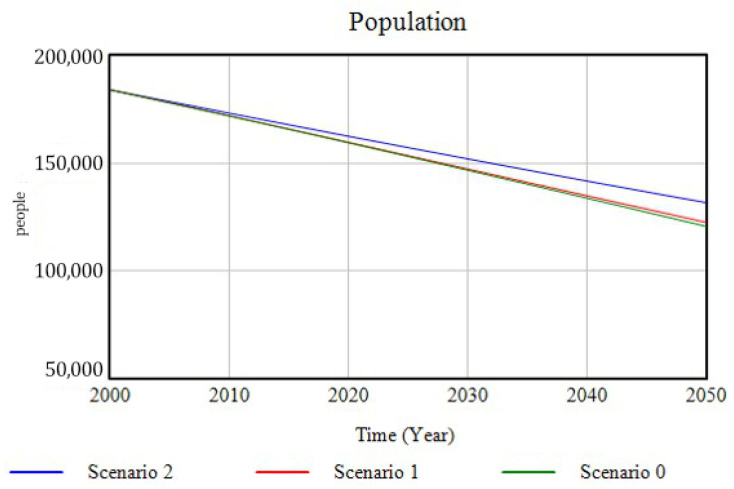
Population evolution for the three scenarios simulated over the period 2000–2500.

Demographic changes in the population are more pronounced for Scenario 2 compared to the previous ones. In this case, births increase due to the influence of employment policies, assuming a 10% increase in the employment rate in the study area (Figure 12). Mortalities, in contrast, are below their previous levels, due to the decrease in the hospital morbidity rate as a result of the drastic reduction in air pollution and its associated diseases, due to the emission control policies established.

#### 3.1.3. Ecosystem Services

**Figure 13:**
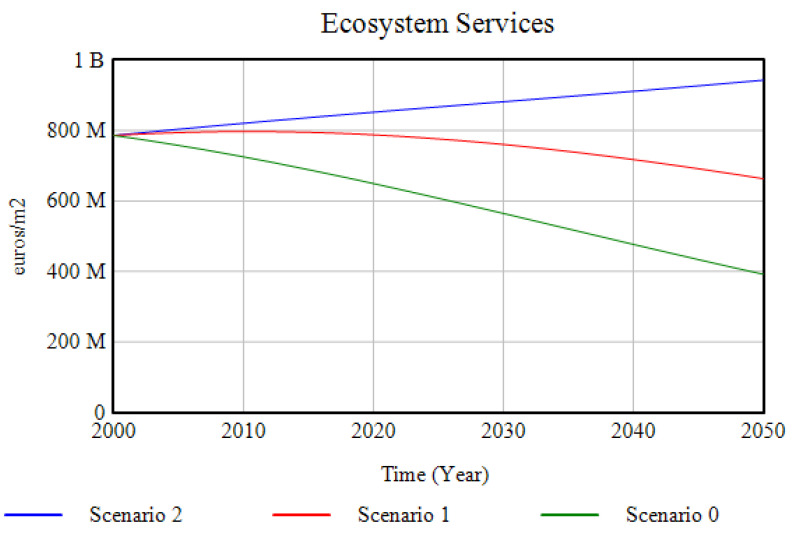
Ecosystem services evolution for the three simulated scenarios over the period 2000–2050.

The evolution of the production of ecosystem services shows a parallel progression to that of the ecosystems in the study area, highlighting its evident strong dependence on the latter (Figure 13). Only in Scenario 2, through the investment in ecosystem restoration and green and blue infrastructure, an increase in the generation of these services is possible.

#### 3.1.4. Economic System

**Figure 14:**
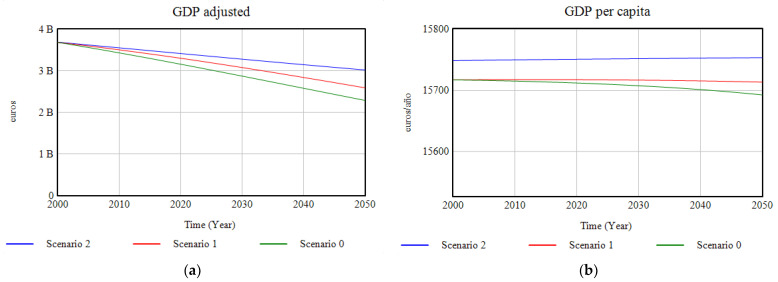
(**a**) Graphic results of the modelling of the adjusted GDP; (**b**) results of the modelling of the GDP per capita, in the period 2000–2050.

The repercussions of the implementation of policies at various levels in this simulation on the main economic indicators (GDP and GDP per capita) are a much steeper downward curve for the progression of adjusted GDP between 2000–2050, due to the economic contribution to Santander of the production of ecosystem services (Figure 14).

#### 3.1.5. Pollution

**Figure 15:**
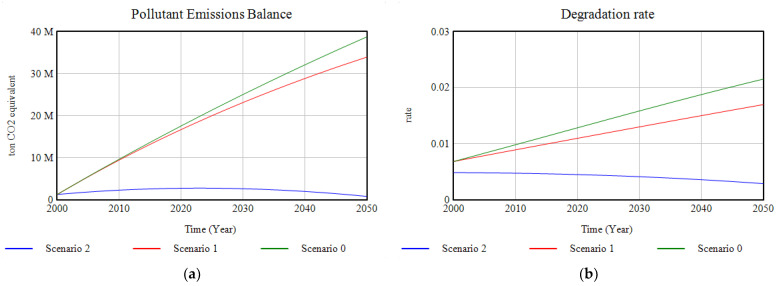
(**a**) Graphic results of the modelling of the pollutant emissions; (**b**) graphic results of the modelling of the ecosystems’ degradation rate, in the period 2000–2050.

Pollution levels due to greenhouse gases emissions show a decrease compared to the baseline scenario due to the increase in the absorption capacity of ecosystems (Figure 15). However, it is in Scenario 2, due to the implementation of environmental policies to reduce emissions, that the drastic reduction in atmospheric pollution, trending towards zero, means that the trend in the rate of degradation of ecosystems is reversed towards a decreasing trend.

#### 3.1.6. Quality-of-Life Indicators

**Figure 16:**
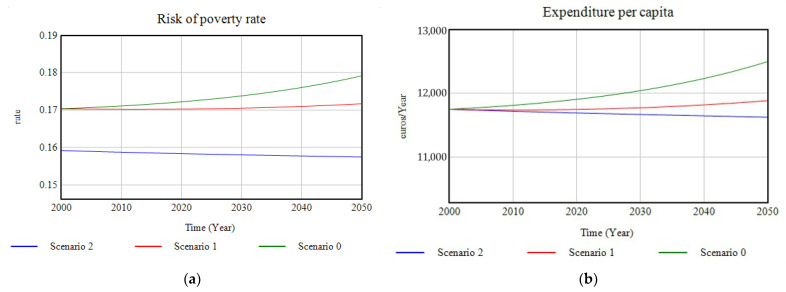
(**a**) Progression of the risk of poverty rate; (**b**) graphic representation of the results of the evolution of the expenditure per capita for the three simulated scenarios in the period 2000–2050.

Concerning the quality-of-life indicators, the graphs presenting the dimensions related to this study, and set out in the Stiglitz–Sen–Fitoussi report, are presented. As an indicator of the material living conditions dimension, first, the evolution of the rate of the population at risk of poverty has been modelled (Figure 16a), which in Scenario 0 experiences exponential growth up to 2050, while investment in Scenario 1 manages to stabilise this growth and, in Scenario 2, due to the complementary action of investment and the policies implemented, it is possible to obtain lower values for this rate and a decreasing trend. Conversely, as it is shown in Figure 16b the variable “expenditure per capita”, which represents the savings that the production of certain ecosystem services represents in household expenditure in Santander, shows an upward trend in the non-action scenario, while its evolution is parallel to that of ecosystem services in the other scenarios, i.e., the higher the production of ecosystem services such as food supply or climate regulation capacity, the lower the expense.

**Figure 17:**
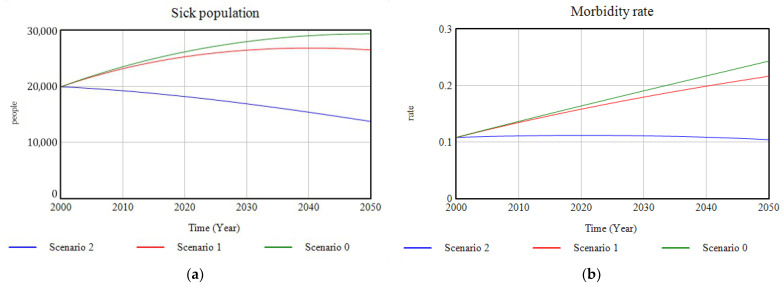
(**a**) Graphic representation of the evolution of sick population; (**b**) graphic results of the evolution of the morbidity rate for the three cases of study, in the period 2000–2050.

**Figure 18:**
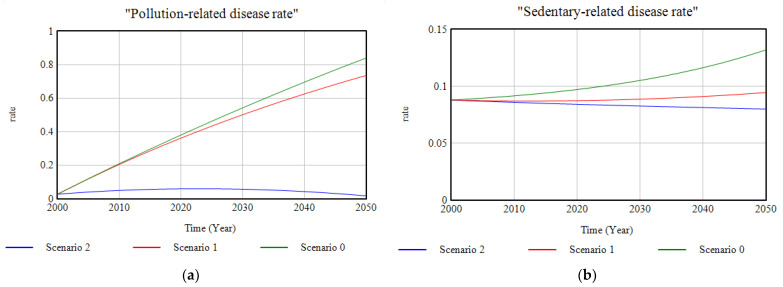
(**a**) Graphic representation of the progression of the pollution-related disease rate; (**b**) graphic representation of the sedentary-related disease rate for the three cases of study modelled, in the period 2000–2050.

Regarding the dimension related to the health state of the population the model provides a graph of the sick population in which can be observed (Figure 17a) how, compared to the growing trend of Scenario 0, investment in ecosystems and green infrastructure implies a decrease in the rate of hospital morbidity (Figure 17b) caused fundamentally by the decrease in the rate of illness due to sedentary habits as a result of the change in the lifestyle of the population due to the availability of more natural spaces which promote physical activity; and, conversely, due to the decrease in the rate of pollution-related illnesses (Figure 18), as a result of the reduction in emissions of certain polluting substances into the atmosphere, such as SSs, NOx, SOx, etc. For Scenario 2, the complementarity of the proposed investment with the set of environmental policies on emissions reduction is a key factor in improving the health status of the inhabitants of the study area.

**Figure 19:**
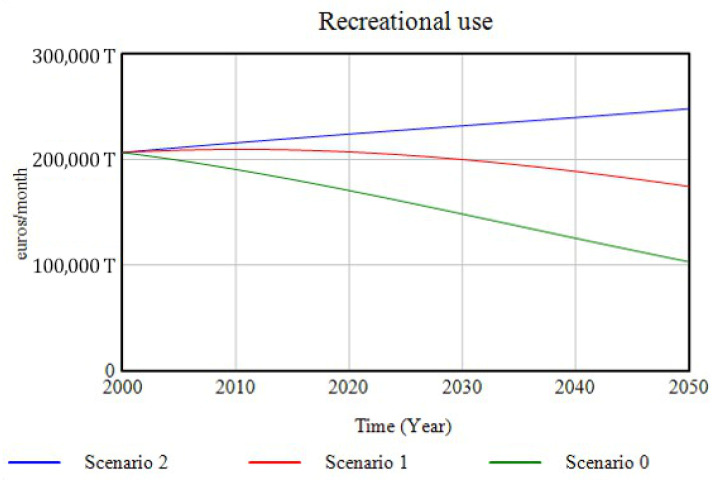
The progression of the ecosystem service “recreational use” for the three simulated scenarios over the period 2000–2050.

The dimension related to leisure and social relations is represented in this model through the variable corresponding to the ecosystem service “recreational use”, which shows, as it can be seen in Figure 19, the evolution for the different scenarios, parallel to that undergone by the ecosystems. For Scenario 2, the spaces allocated to the social and recreational activities of ecosystems and green infrastructures undergo a positive progression, bringing an increased economic benefit to the city. 

**Figure 20:**
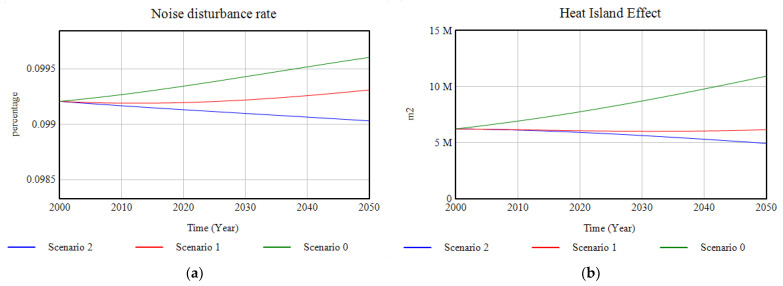
(**a**) Graphic representation of the noise disturbance rate; (**b**) graphic representation of the results of the modelling of the heat island effect for the cases of study, in the period 2000–2050.

In terms of the quality-of-life indicators relating to the surroundings and the environment, the following results are obtained. The rate of noise pollution is reduced due to investment in ecosystem restoration and the incorporation of green infrastructures in the city, which act as natural barriers to acoustic pollution, but it is in Scenario 2, in conjunction with transport policies that reduce the noise pollution emitted by vehicle traffic, that this rate shows a downward trend. The heat island effect (Figure 20b) in the city of Santander shows a similar progression to the evolution of the impervious surface and is dependent on the heat regulation capacity of the city due to the implementation of green infrastructure and the presence of ecosystems, achieving a reduction, by the year 2050, of up to half the value predicted for the most unfavourable scenario.

### 3.2. Economic Assessment

The following tables show the numerical values of investments and adjusted GDP for different years within the study period 2000–2050 for scenarios 1 and 2, provided by VENSIM for each simulation.

Table 3 and Table 4 show the investment in green infrastructures and policies and its effect on Santander’s GDP compared to a base case (Scenario 0), for different years within the study period. With minor investments (less than EUR 35 million per year), the increase of GDP is relevant. These results show that EUR 1 spent as green infrastructure and policies translates into EUR 100 increase in GDP during all the period considered.

From the results shown in Table 5, based on the non-action scenario, the investment of an annual 2% of Santander’s GDP in ecosystem restoration and green infrastructure implies an increase in GDP by 2050 of 13.24%, whereas, for Scenario 2, the complementation of the investment with the set of environmental and socio-economic policies implies an increase in adjusted GDP of 32.04%.

Based on these results, and the assumptions considered in the modelling, the cost-effectiveness of the investment and policies implemented can be considered to be favourable.

## 4. Conclusions

The model presented in this work exemplifies the feasibility of representing, by means of an SD model, the complex dynamics of a region as Santander Bay, covering economic, social, and environmental spheres. The results obtained from this work allow visualising the complex feed-back mechanisms connecting direct income factors with quality of life through environmental and health drivers.

Nevertheless, as quantitative and qualitative limitations have emerged in the calibration process, that may limit the exploitation of the model for further analysis, additional research is required to improve the accuracy of the parameters.

The model itself can greatly benefit from the extensive review of variables interaction and their inherent degrees of freedom. This approach may result, not only in results that are more accurate, but also on a more general model, applicable to different scenarios and study sites. The precise quantification of the phenomena can greatly benefit from additional fieldwork, but at the present state, the consistent analysis developed for the alternatives allow understanding the existing mechanisms may drive the evolution.

Finally, it is concluded that, under the assumptions considered in the modelling and its calibration and derived from the results obtained for the three simulation scenarios, conservation and regeneration of ecosystems and green and blue infrastructure, and the implementation of environmental and socio-economic policies, is economically, environmentally, and socially profitable. Results show that EUR 1 spent in these policies and projects could produce up to EUR 100 as a benefit provided as an increase in GDP. The annual investment of 2% of Santander’s GDP leads to the progress of the local economy in up to 32%, while resulting in an improvement in the quality of life of the inhabitants of the study area.

Although these results are limited by the constraints defined above, this study shows that SD models are a tool to be considered by government managers to analyse their actions and policies. In this sense, SD models allow to analyse potential actions from a multiple point of view (economic, social, environmental) considering the different systems that share the city (population, economy, ecosystems, etc.). Moreover, the model can be easily transfer to a different urban area where the fieldwork may help to characterise the problems associated to the site.

The main advantage of SD models over other tools currently being considered for the socioeconomic analysis of actions and policies is that they allow for the incorporation of the behaviour of different systems and their interactions, analysing in a more complete way, the effects of actions and policies on each of them.

The application shown in this study shows that SD models cannot only have a place in the management of urban areas, but it also raises possibilities for use in other areas and current risks. For example, the use of dynamic systems can also be considered as a good option for the analysis of climate change adaptation actions in areas that may be at risk.

## Figures and Tables

**Table 1:** Ecosystems present in the study area and its associated ecosystem services (based on [43]).

	Forests and Heathland	Croplands	Coastal Ecosystems	Inland Waters, Wetlands and Estuaries	Grasslands and Pastures
Food	X	X	X	X	X
Materials	X	X			X
Extreme events	X		X	X	
Air quality	X	X	X	X	X
Water purification	X	X		X	X
Climate regulation	X	X		X	X
Hydrological cycle management	X	X	X	X	X
BioControl	X	X	X	X	X
Pollination	X	X			X
Recreational use	X	X	X	X	X

**Table 2:** Green Infrastructure present in the study area and its associated ecosystem services (based on [43]).

	Green Urban Areas	SUDS	Urban Gardens	Green Corridors
Food			X	
Materials			X	
Extreme events	X	X		X
Air quality	X		X	X
Water purification	X	X	X	X
Climate regulation	X	X	X	X
Hydrological cycle management	X	X	X	X
BioControl	X	X	X	X
Pollination	X	X	X	X
Recreational use	X		X	X

**Table 3:** Numerical results of investment values and adjusted GDP variation for Scenario 1.

	2010	2020	2030	2040	2050
Investment (EUR mill.)	35.03	33.00	30.77	28.38	25.89
GDP (EUR mill.)	3503.01	3300.23	3077.48	2838.98	2589.35

**Table 4:** Numerical results of investment values and adjusted GDP variation for Scenario 2.

	2010	2020	2030	2040	2050
Investment (EUR mill.)	35.51	34.14	32.78	31.46	30.19
GDP (EUR mill.)	3551.24	3414.10	3278.43	3146.13	3019.27

**Table 5:** Percentages of the increase in adjusted GDP as a result of the actions carried out in each simulation scenario.

	2010	2020	2030	2040	2050
Case 0–Case 1	2.13	4.53	7.18	10.08	13.24
Case 0–Case 2	3.54	8.14	14.18	21.99	32.04
Case 1–Case 2	1.38	3.45	6.53	10.82	16.60

## Data Availability

Not applicable.
